# Supplementary material for: The organisation of primary health care service delivery for non-communicable diseases in Nigeria: A case-study analysis
Source: PLOS Glob Public Health. 2022 Jul 1;2(7):e0000566. doi: 10.1371/journal.pgph.0000566 (PMC10021956; doi:10.1371/journal.pgph.0000566)
Supplement: S1 Annex — (DOCX) [file pgph.0000566.s001.docx]

**HEALTH FACILITY SUMMARIES**

**Table 1: Summary of PHC personnel (doctors, nurses and community health workers)**

| **Health Facility** | **Regional Location** | **Personnel** | | | | | | | | | |
| --- | --- | --- | --- | --- | --- | --- | --- | --- | --- | --- | --- |
|  |  | **Doctors** | | **Nurse/Midwives** | | **CHO** | | **CHEW** | | **JCHEW** | |
|  |  | **Total** | **part-time** | **Total** | **part-time** | **Total** | **part-time** | **Total** | **part-time** | **Total** | **part-time** |
| PHC1 | North | 1 | 1 | 4 | 2 | 0 | 0 | 3 | 0 | 9 | 8 |
| PHC2 | North | 1 | 1 | 1 | 0 | 1 | 0 | 3 | 0 | 0 | 0 |
| PHC3 | North | 0 | 0 | 5 | 1 | 3 | 0 | 7 | 2 | 0 | 0 |
| PHC4 | North | 0 | 0 | 1 | 0 | 0 | 0 | 9 | 4 | 9 | 5 |
| PHC5 | North | 0 | 0 | 0 | 0 | 1 | 0 | 7 | 5 | 3 | 2 |
| PHC6 | North | 0 | 0 | 0 | 0 | 1 | 0 | 5 | 3 | 8 | 4 |
| PHC7 | North | 0 | 0 | 0 | 0 | 1 | 0 | 2 | 1 | 3 | 3 |
| PHC8 | South | 4 | 0 | 4 | 2 | 2 | 0 | 9 | 0 | 0 | 0 |
| PHC9 | South | 3 | 0 | 7 | 2 | 1 | 0 | 2 | 1 | 1 | 1 |
| PHC10 | South | 4 | 0 | 9 | 3 | 4 | 0 | 3 | 0 | 2 | 2 |
| PHC11 | South | 2 | 0 | 11 | 1 | 0 | 0 | 13 | 7 | 1 | 0 |
| PHC12 | South | 1 | 0 | 3 | 0 | 0 | 0 | 4 | 1 | 2 | 0 |
| PHC13 | South | 0 | 0 | 4 | 1 | 0 | 0 | 6 | 3 | 1 | 0 |

# PHC 1

**Health facility description and personnel**

This is a Comprehensive Health Centre situated at the Abuja Municipal Area Council. It currently serves a population of about 25,375 as against recommended 10,000 – 20,000 population coverage. It has a part time Medical Doctor who is on the mandatory one-year national service. Eight out of the nine Junior Community Health Extension Worker (JCHEW) are either volunteers or contract staff (Table 1). A CHEW designated as the Officer-in-Charge (OIC) oversees the PHC administration. In order to retain the healthcare workers and sustain the running of the PHC, the OIC uses internally generated revenues (such as gain made from drug sales and other services provided to patients). Some of the volunteer/contract staff claim to have worked in that capacity for a decade.

**Patients access to facility**

The PHC operates a 24-hour service and patients can access the facilities by a follow up appointment or walk in whenever there is a need. Apart from community mental health services, all basic primary health care services such as immunization and treatment of minor ailments are being offered. Use of paper file is the only means of keeping patients’ records and the patients’ information are kept by the facility only. Apart from labour-ward/maternity beds, facility has 8 beds for in-patient admission and attended to 309 patients in the month preceding the assessment. Of these patients, 4 were hypertensive and 1 was diabetic.

**Basic amenities**

The facilities main supply of electricity is the national grid but uses a generator as back up electricity source. The facility experiences more than 2 hours interruption of electricity supplies. Water piped within the facility is the main source of water and there is a functioning flushed toilet type in the PHC. Final disposal of waste is done by open burning. The facility has no functional computer, dedicated landline telephone or private telephone. However, individual staff has their personal phone through which they have access to internet or private email.

**Service delivery for NCDs**

The CHWs screens, diagnoses, prescribes and dispenses initial treatment for high blood pressure and diabetes. It also prescribes follow-up treatment, monitors and provide long term care for these groups of patients. While CHWs are involved in the care of patients with hypertension and diabetics, none has received training for management of NCDs within the last 2 years. The facility also refers NCDs cases to the nearest secondary health facilities for some reasons such as lack of BP control despite 3 drug therapy. However, there is no ambulance or vehicle to convey referred patients. So, patients are responsible for how they transport themselves to the referral centres. The facility also has no national guideline or treatment algorithm for the management of NCDs.

Though there is no information, education and communication (IEC) materials available, the facility conducts counselling and education for patients and their relatives on NCDs risk factors. The facility does not also receive any community support such as finances concerning NCDs services.

**Basic NCDs equipment, diagnostic kits and drugs**

The facility has at least one functional sphygmomanometer, stethoscope, adult weighing scale, glucometer, thermometer and measuring tape. There are also test kits to check for urine glucose, protein, ketone bodies but not for albumin.

The facility also has in stock some basic NCDs medications such as anti-hypertensives (calcium-channel blockers, ACE-Inhibitors & centrally acting agents), anti-diabetics (metformin) and bronchodilators (salbutamol)

**Supply chain structures and transportation**

The Officer-in-Charge (a CHEW) is the principal person responsible for managing the ordering of the facility’s medical supply. The national medical store is the main source for routine pharmaceutical supplies and the facility has to arrange the mechanisms of transportation to deliver product to the facility. For routine medications, private suppliers deliver stock to the facility and the typical time interval between product ordering and delivery is usually less than 2 weeks. Pull distribution, in which the facility make request, is the main mechanism of determining the facility’s resupply need and there is no specific formula for determining the quantity needed for resupply.

# PHC 2

**Health facility description and personnel**

This is a Primary Health Clinic. It currently serves a population of about 10,233 as against recommended 2,000 – 5,000 population coverage. It has a part time Medical Doctor who is on the mandatory one-year national service. This facility is overseen by the Nurse who serves as the Officer-in-Charge. Other permanent staff are 1 Community Health Officer (female) and 3 Community Health Extension Worker (CHEW) (Table 1). The facility has three Junior Community Health Extension Worker (JCHEW) who are either volunteers or contract staff. The OIC uses internally generated revenues (such as gain made from drug sales and other services provided to patients) and encourages voluntary contribution from the salary of the permanent staff in order retain the volunteer healthcare workers for the sustenance of health services at the PHC.

**Patients access to facility**

The PHC operates an 8-hour services and patients can access the facilities by a follow up appointment or walk in whenever there is a need. Being in a remote setting, the already over-burdened staff has made some internal arrangement to be able to provide additional hours of service that will cater for labour and delivery cases beyond regular work hours. All range of basic primary health care services such as maternal and child health services, immunization and treatment of minor ailments are being offered. Paper file is the only means of keeping patients’ records and the patients’ information are kept by the facility only. Apart from labour-ward/maternity beds, the facility has no bed for in-patient admission. A total of 163 patients were attended to in the month preceding assessment. None of these patients were hypertensive or diabetic.

**Basic amenities**

The facility uses generator as the main source of electricity and has no back up electricity source***. The staff mentioned that there was no provision for electrical wiring when the facility was built. It was by the efforts of the OIC that electrical wiring was provided for some part of the building to conduct some laboratory tests.*** The facility experiences more than 2 hours interruption of electricity supplies. Water piped within the facility is the main source of water and there is a functioning flushed toilet type in the PHC. Final disposal of waste is done by removal from the site. The facility has no functional computer, dedicated landline telephone, private telephone, internet facility or access to email.

**Service delivery for NCDs**

The facility screens, diagnose, prescribe initial treatment for high blood pressure and diabetes. However, it **does not dispense any drugs for diabetics or hypertension**. Staff occasionally provides follow-up treatment and monitors hypertension patients. CHWs are involved in the care of patients with hypertension but none has received training for management of NCDs within the last 2 years. The facility refers all NCDs cases to the nearest secondary health facility located about 10 kms away for every possible reason. There is no ambulance or vehicle to convey referred patients. So, patients are responsible for how they transport themselves to the referral centres. The facility also has no national guideline or treatment algorithm for the management of NCDs. Though there is no information, education and communication (IEC) materials available, the facility conducts counselling and education for patients and their relatives on NCDs risk factors. The facility does not also receive any community support concerning NCDs services.

**Basic NCDs equipment, diagnostic kits and drugs**

The facility has at least one functional sphygmomanometer, stethoscope, adult weighing scale, glucometer, thermometer and measuring tape. There are also test kits to check for urine glucose, protein, ketone bodies but not for albumin.

The facility also has no NCDs medications in stock. Patients that are being monitored are advised to visit a pharmacy to refill their drugs

**Supply chain structures and transportation**

The Officer-in-Charge (a Matron) is the principal person responsible for managing the ordering of the facility’s medical supply. private pharmaceutical companies are the main source for routine pharmaceutical supplies and the suppliers deliver the consignment directly to the facility. The same also applies to routine medications, private suppliers deliver stock to the facility and the typical time interval between product ordering and delivery is usually less than 2 weeks. Pull distribution, in which the facility make request, is the main mechanism of determining the facility’s resupply need and there is no specific formula for determining the quantity needed for resupply.

# PHC 3

**Health facility description and personnel**

This is a Comprehensive Health Centre. It currently serves a population of about 60,375 as against recommended 10,000 – 20,000 population coverage. Despite being a facility that attend to a huge population of patient, it has no Medical Doctor. It also has no Junior Community Health Extension Worker (JCHEW) but has 7 CHEWs of which 2 are part time staff, 3 Community Health Officer (CHO) and 5 nursing staff (Table 1). The Officer-in-Charge (OIC), who oversees the PHC administration is a Matron. In order retain the healthcare workers and sustain the running of the PHC, the OIC uses internally generated revenues (such as gain made from drug sales and other services provided to patients).

The Matron in charge of the facility and the 3 CHO are involve in direct consultation and management of patient. It was also observed that the Matron also directly dispense (sells) personal drugs (outside facility stocks) to patients she attends to in her consulting room.

**Patients access to facility**

The PHC operates a 24-hour services and patients can access the facilities by a follow up appointment or walk in whenever there is a need. All basic primary health care services such as immunization and treatment of minor ailments are being offered by the facility. Use of paper file is the only means of keeping patients’ records and the patients’ information are kept by the facility only. Apart from labour-ward/maternity beds, facility has 8 beds for in-patient admission and attended to 3456 patients in the month preceding assessment. Of these patients, 5 were hypertensive and 5 were diabetic.

**Basic amenities**

The facilities main supply of electricity is the national grid but uses a generator as back up electricity source. The facility experiences more than 2 hours interruption of electricity supplies. Water piped within the facility is the main source of water and there is a functioning flushed toilet type in the PHC. Final disposal of waste is done by open burning. The facility has no functional computer, dedicated landline telephone or private telephone. However, individual staff has their personal phone through which they have access to internet or private email.

**Service delivery for NCDs**

The facility screens, diagnose, prescribe and dispense initial treatment for high blood pressure and diabetes. It also prescribes follow-up treatment, monitors and provide long term care for the hypertensive but not for patients. While CHWs are involved in the care of patients with hypertension and diabetics, no staff of the facility has received training for management of NCDs within the last 2 years. The facility also refers NCDs cases to the nearest secondary health facilities for reasons such as patients’ preference or symptoms thought to be likely caused by high blood pressure. However, there is no ambulance or vehicle to convey referred patients. So, patients are responsible for how they transport themselves to the referral centres. There are three secondary health facilities that patients are usually referred to – these are located about 10 – 30 kms away.

The facility has no national guideline or treatment algorithm for the management of NCDs.

Though there is no information, education and communication (IEC) materials available, the facility conducts counselling and education for patients and their relatives on NCDs risk factors. The facility does not also receive any community support concerning NCDs services.

**Basic NCDs equipment, diagnostic kits and drugs**

The facility has at least one functional sphygmomanometer, stethoscope, adult weighing scale, glucometer, thermometer and measuring tape. There are also test kits to check for urine glucose, protein, ketone bodies but not for albumin.

The facility also has in stock some basic NCDs medications such as anti-hypertensives (Thiazide, calcium-channel blockers, Angiotensin receptor blockers, ACE-Inhibitors & centrally acting agents), antiplatelet (low dose aspirin) anti-diabetics (metformin) and bronchodilators (salbutamol)

**Supply chain structures and transportation**

Though the Pharmacist is the principal person responsible for managing the ordering of the facility’s medical supply, ***the Officer-in-Charge (a Matron) also bear significant influence. The Pharmacist also expressed displeasure concerning a parallel and illegitimate supply chain that operates within the facility by the support of the Matron in-charge.*** The national medical store is the main source for routine pharmaceutical supplies and the facility has to arrange the mechanisms of transportation to deliver product to the facility. For routine medications, private suppliers deliver stock to the facility and the typical time interval between product ordering and delivery is usually less than 2 weeks. Pull distribution, in which the facility make request, is the main mechanism of determining the facility’s resupply need and there is no specific formula for determining the quantity needed for resupply.

# PHC 4

**Health facility description and personnel**

This is a Comprehensive Health Centre. It currently serves an estimated population of about 8,085 compared with the recommended 10,000 – 20,000 population coverage. The facility has only 1 nurse but has no Medical Doctor. Five of the 9 Junior Community Health Extension Workers (JCHEW) and 4 out of the 9 Community Health Extension Workers (CHEWs) are part time staff (Table 1). The Officer-in-Charge (OIC) of the facility is a CHEW. The part time staff are retained by the OIC who uses internally generated revenues (such as gain made from drug sales and other services provided to patients) in order to sustain the day-to-day running of the facility. Being a facility that also operated a performance-based financing (PBF) scheme, bonuses obtained from the scheme also add to engaging contract staff.

**Patients access to facility**

The PHC operates a 24-hour services and patients can access the facilities by a follow up appointment or walk in whenever there is a need. All basic primary health care services such as immunization and treatment of minor ailments are being offered by the facility. Use of paper file is the only means of keeping patients’ records and the patients’ information are kept by the facility only. Apart from labour-ward/maternity beds, facility has 8 beds for in-patient admission and attended to 1127 patients in the month preceding assessment. Of these patients, 3 were hypertensive and 4 were diabetic.

**Basic amenities**

The facilities main supply of electricity is the national grid but uses a generator as back up electricity source. The facility experiences more than 2 hours interruption of electricity supplies. Water piped within the facility is the main source of water and there is a functioning flushed toilet type in the PHC. Final disposal of waste is done by open burning. The facility has no functional computer, dedicated landline telephone or private telephone. However, individual staff has their personal phone through which they have access to internet or private email.

**Service delivery for NCDs**

While the facility offers every service for cases of hypertension, only screening and diagnosis, is offered for cases of diabetes. CHWs are involved in the care of patients with hypertension and diabetics but no staff of the facility has received training for management of NCDs within the last 2 years. The facility also refers NCDs cases to the nearest secondary health facilities for reasons such as HCW’s preference. Absolute BP levels (e.g. 150/100mmHg) or Lack of BP control despite 3-drug therapy. The facility has no ambulance or vehicle to convey referred patients. So, patients are responsible for how they transport themselves to the referral centres. The only secondary health facilities that patients are usually referred to is located 10 kms away.

The facility has no national guideline or treatment algorithm for the management of NCDs.

Though there is no information, education and communication (IEC) materials available, the facility conducts counselling and education for patients and their relatives on NCDs risk factors. The facility does not also receive any community support concerning NCDs services.

**Basic NCDs equipment, diagnostic kits and drugs**

The facility has at least one functional sphygmomanometer, stethoscope, adult weighing scale, glucometer, thermometer and measuring tape. There are also test kits to check for urine glucose, protein, ketone bodies but not for albumin. ***The facility has no refrigerator for preserving supplies.***

The facility also has in stock some basic NCDs medications such as anti-hypertensives (Thiazide, calcium-channel blockers, diuretics, ACE-Inhibitors & injectable vasodilators), bronchodilators (salbutamol) but no anti-diabetics drugs.

**Supply chain structures and transportation**

The officer-in-charge (CHEW) is the principal person responsible for managing the ordering of the facility’s medical supply. Private sources are the main source for routine pharmaceutical supplies and the facility has to arrange the mechanisms of transportation to deliver product to the facility. For routine medications, private suppliers deliver stock to the facility and the typical time interval between product ordering and delivery is usually less than 2 weeks. Both pull and push distribution, in which the facility make request, or it is supplied by higher level, are the main mechanism of determining the facility’s resupply need and there is no specific formula for determining the quantity needed for resupply.

# PHC 5

**Health facility description and personnel**

This is a Comprehensive Health Centre. It currently serves an estimated population of about 12,118 compared with the recommended 10,000 – 20,000 population coverage. The facility has no Medical Doctor or a nurse. Two of the 3 Junior Community Health Extension Workers (JCHEW) and 5 out of the 7 Community Health Extension Workers (CHEWs) are part time staff (Table 1). There is one Community Health Officer who is also the Officer-in-Charge (OIC) of the facility. The part time staff are retained by the OIC through internally generated revenues (such as gain made from drug sales and other services provided to patients) in order to sustain the day-to-day running of the facility. Bonuses from the performance-based financing (PBF) scheme, are also used to pay the engaged contract staff.

**Patients access to facility**

The PHC operates a 24-hour services and patients can access the facilities by a follow up appointment or walk in whenever there is a need. All basic primary health care services such as immunization and treatment of minor ailments are being offered by the facility. Use of paper file is the only means of keeping patients’ records and the patients’ information are kept by the facility only. Apart from labour-ward/maternity beds, facility has 14 beds for in-patient admission and attended to 1064 patients in the month preceding assessment. Of these patients, 64 were hypertensive and 15 were diabetic.

**Basic amenities**

The facilities main supply of electricity is the national grid but uses a generator as back up electricity source. The facility experiences more than 2 hours interruption of electricity supplies. Water piped within the facility is the main source of water and there is a functioning flushed toilet type in the PHC. Final disposal of waste is done by burning in an incinerator. The facility has a functional computer and a private telephone but no dedicated landline telephone.

**Service delivery for NCDs**

While the facility offers every service for cases of hypertension, only screening and diagnosis, is offered for cases of diabetes. CHWs are involved in the care of patients with hypertension and diabetics but no staff of the facility has received training for management of NCDs within the last 2 years. The facility also refers NCDs cases to the nearest secondary health facilities for reasons such as symptoms thought likely to be caused by high BP, HCW’s preference, Absolute BP levels (e.g. 160/100mmHg) or lack of BP control despite 3-drug therapy. The facility has no ambulance or vehicle to convey referred patients. So, patients are responsible for how they transport themselves to the referral centres. There is only secondary health facilities that patients are referred to.

The facility has no national guideline or treatment algorithm for the management of NCDs.

Though there is no information, education and communication (IEC) materials available, the facility conducts counselling and education for patients and their relatives on NCDs risk factors. The facility does not also receive any community support concerning NCDs services.

**Basic NCDs equipment, diagnostic kits and drugs**

The facility has at least one functional sphygmomanometer, stethoscope, adult weighing scale, glucometer, thermometer and measuring tape. There are also test kits to check for urine glucose, protein, ketone bodies but not for albumin.

The facility also has in stock some basic NCDs medications such as anti-hypertensives (calcium-channel blockers, diuretics, ACE-Inhibitors & centrally acting agents), bronchodilators (salbutamol) and anti-diabetics drugs.

**Supply chain structures and transportation**

The officer-in-charge (a CHO) is the principal person responsible for managing the ordering of the facility’s medical supply. Private sources are the main source for routine pharmaceutical supplies and the facility has to arrange the mechanisms of transportation to deliver product to the facility. For routine medications, private suppliers deliver stock to the facility and the typical time interval between product ordering and delivery is usually less than 2 weeks. Facility’s resupply quantity is determined by the use of a specific formula.

# PHC 6

**Health facility description and personnel**

This is a Comprehensive Health Centre. It currently serves an estimated total ward population of 9033 and catchment population of about 7395 compared with the recommended 10,000 – 20,000 population coverage. The facility has no Medical Doctor or a nurse. 4 of the 8 Junior Community Health Extension Workers (JCHEW) and 3 out of the 5 Community Health Extension Workers (CHEWs) are part time staff. There is one Community Health Officer who is also the Officer-in-Charge (OIC) of the facility. The part time staff are retained by the OIC through internally generated revenues (such as gain made from drug sales and other services provided to patients) in order to sustain the day-to-day running of the facility. Bonuses from the performance-based financing (PBF) scheme, are also used to pay the engaged contract staff.

This is also one of the facilities where the practical approach to care kit (PACK) programme was piloted in the country.

**Patients access to facility**

The PHC operates a 24-hour services and patients can access the facilities by a follow up appointment or walk in whenever there is a need. All basic primary health care services such as immunization and treatment of minor ailments are being offered by the facility. Use of paper file is the only means of keeping patients’ records and the patients’ information are kept by the facility only. Apart from labour-ward/maternity beds, facility has 16 beds for in-patient admission and attended to 402 patients in the month preceding assessment. Of these patients, 12 were hypertensive and 16 were diabetic.

**Basic amenities**

The facilities main supply of electricity is the national grid but uses a generator as back up electricity source. The facility experiences more than 2 hours interruption of electricity supplies. Water piped within the facility is the main source of water and there is a functioning flushed toilet type in the PHC. Final disposal of waste is done by removing them offsite. The facility has a functional computer and a private telephone but no dedicated landline telephone.

**Service delivery for NCDs**

The facility screens, diagnose and monitor patients with high blood pressure and high blood but only prescribe and dispense drugs for high blood pressure. sugar. CHWs are mainly responsible for patient management including the care of patients with hypertension and diabetics but none of them has received training for management of NCDs within the last 2 years. The facility also refers NCDs cases to the nearest secondary health facilities for reasons such as symptoms thought likely to be caused by high BP, HCW’s preference, Absolute BP levels (e.g. 160/100mmHg) or patients’ preference. The facility has no ambulance or vehicle to convey referred patients. Patients and their relatives are responsible for how they transport themselves to the referral centres. There is only one secondary health facilities that patients are usually referred to within 10km of the facility.

The facility has no national guideline but utilizes the PACK guideline for the management of NCD. There is no information, education and communication (IEC) materials available on NCDs but the facility conducts counselling and education for patients and their relatives on NCDs risk factors.

**Basic NCDs equipment, diagnostic kits and drugs**

The facility has at least one functional sphygmomanometer, stethoscope, adult weighing scale, glucometer, thermometer and measuring tape. There are also test kits to check for urine glucose, protein, ketone bodies but not for albumin.

The facility also has in stock some basic NCDs medications such as anti-hypertensives (calcium-channel blockers, vasodilators & centrally acting agents), bronchodilators (salbutamol) but no anti-diabetics drugs.

**Supply chain structures and transportation**

The officer-in-charge (a CHO) is the principal person responsible for managing the ordering of the facility’s medical supply. Private sources are the main source for routine pharmaceutical supplies and the facility has to arrange the mechanisms of transportation to deliver product to the facility. For routine medications, private suppliers deliver stock to the facility and the typical time interval between product ordering and delivery is usually less than 2 weeks. Facility’s resupply quantity is determined using a specific formula.

# PHC 7

**Health facility description and personnel**

This is a Comprehensive Health Centre. It currently serves an estimated total ward population of 10382 and catchment population of about 4662 compared with the recommended 10,000 – 20,000 population coverage. The facility has no Medical Doctor or a nurse. All of the 3 Junior Community Health Extension Workers (JCHEW) and 1 out of the 1 Community Health Extension Workers (CHEWs) are part time staff (Table 1). There is one Community Health Officer who is also the Officer-in-Charge (OIC) of the facility. The part time staff are retained by the OIC through internally generated revenues (such as gain made from drug sales and other services provided to patients) in order to sustain the day-to-day running of the facility. Bonuses from the performance-based financing (PBF) scheme, are also used to pay the engaged contract staff. This is also one of the facilities where the practical approach to care kit (PACK) was piloted in the country.

**Patients access to facility**

The PHC operates a 24-hour services and patients can access the facilities by a follow up appointment or walk in whenever there is a need. All basic primary health care services such as immunization and treatment of minor ailments are being offered by the facility. Use of paper file is the only means of keeping patients’ records and the patients’ information are kept by the facility only. Apart from labour-ward/maternity beds, facility has 10 beds for in-patient admission and attended to 384 patients in the month preceding assessment. Of these patients, 3 were hypertensive and 1 were diabetic.

**Basic amenities**

The facilities main supply of electricity is the national grid but uses a generator as back up electricity source. The facility experiences more than 2 hours interruption of electricity supplies. Water piped within the facility is the main source of water and there is a functioning flushed toilet type in the PHC. Final disposal of waste is done by removing them offsite. The facility has a functional computer and a private telephone but no dedicated landline telephone.

**Service delivery for NCDs**

The facility screens, diagnose and monitor patients with high blood pressure and high blood but only prescribe and dispense drugs for high blood pressure. sugar. CHWs are mainly responsible for patient management including the care of patients with hypertension and diabetics but none of them has received training for management of NCDs within the last 2 years. The facility also refers NCDs cases to the nearest secondary health facilities for reasons such as symptoms thought likely to be caused by high BP, HCW’s preference, Absolute BP levels (e.g. 170/110mmHg) or patients’ preference. The facility has no ambulance or vehicle to convey referred patients. Patients and their relatives are responsible for how they transport themselves to the referral centres. The nearest secondary health facilities that patients are usually referred to is a General Hospital within 15km radius.

The facility has no national guideline but utilizes the PACK guideline for the management of NCD. There is no information, education and communication (IEC) materials available on NCDs but the facility conducts counselling and education for patients and their relatives on NCDs risk factors. The facility does not also receive any community support concerning NCDs services.

**Basic NCDs equipment, diagnostic kits and drugs**

The facility has at least one functional sphygmomanometer, stethoscope, adult weighing scale, glucometer, thermometer and measuring tape. There are also test kits to check for urine glucose, protein, ketone bodies but not for albumin.

The facility also has in stock some basic NCDs medications such as anti-hypertensives (thiazides and Fixed Dose Combinations (e.g. lisinopril-hydrochlorothiazide) but no bronchodilators or anti-diabetics drugs.

**Supply chain structures and transportation**

The officer-in-charge (a CHO) is the principal person responsible for managing the ordering of the facility’s medical supply. Private sources are the main source for routine pharmaceutical supplies and the facility has to arrange the mechanisms of transportation to deliver product to the facility. For routine medications, facility make arrangement for collection and the typical time interval between product ordering and delivery is usually less than 2 weeks. Facility’s resupply quantity is determined using a specific formula.

# PHC 8

**Health facility description and personnel**

This is a Comprehensive Health Centre in the southern region. It currently serves a population of about 62,022 as against recommended 10,000 – 20,000 population coverage. It has 4 full-time Medical Doctor and 4 nurses of which 2 are part-time. Though, there are no Junior Community Health Extension Worker (JCHEW), there are 2 full time Community Health Officers (CHO) and 9 Community Health Extension Worker (CHEW) (Table 1).

This PHC runs a shift duty so that there is at least a medical doctor on duty throughout the 24 hours. It is also referred to as flagship centre where lower level of PHCs can refer patient to.

**Patients access to facility**

The PHC operates a 24-hour services and patients can access the facilities by a follow up appointment or walk in whenever there is a need. All basic primary health care services such as immunization and treatment of minor ailments are being offered within the facility. Use of paper file is the only means of keeping patients’ records and the patients’ information are kept by the facility only. Apart from labour-ward/maternity beds, the PHC have no provision for in-patient’s admission; and attended to 2699 patients in the month preceding assessment. Of these patients, 28 were hypertensive and 2 was diabetic.

**Basic amenities**

The facilities main supply of electricity is the national grid but uses a generator as back up electricity source. The facility experiences more than 2 hours interruption of electricity supplies. Water piped within the facility is the main source of water and there is a functioning flushed toilet type in the PHC. Final disposal of waste is done by removal from offsite by the medical branch of State Waste Management Agency. The facility has a functional computer but no dedicated landline telephone or private telephone. However, individual staff has their personal phone through which they have access to internet or private email.

**Service delivery for NCDs**

The facility screens, diagnose, prescribe and dispense initial and follow up treatment for high blood pressure and diabetes cases. It also provides long term care for these groups of patients. Some staff of the facilities have received in-service training for management of patients with hypertension and diabetics within the last 2 years. CHWs are also involved in the management of NCDs cases. The facility refers NCDs cases to the nearest secondary health facilities for some reasons such as lack of BP control despite 3 drug therapy. There is a functional ambulance available to convey referred patients, at no cost to the patients or their relatives. However, the facility has no national guideline or treatment algorithm for the management of NCDs.

Though there is no information, education and communication (IEC) materials available, the facility conducts counselling and education for patients and their relatives on NCDs risk factors. While the facility does not also receive a direct community support concerning NCDs services, it occasionally partners with a commercial bank to provide NCDs services to the community.

**Basic NCDs equipment, diagnostic kits and drugs**

The facility has at least one functional sphygmomanometer, stethoscope, adult weighing scale, glucometer, thermometer and measuring tape. There are also test kits to check for urine glucose, protein, ketone bodies but not for albumin.

The facility also has in stock some basic NCDs medications such as anti-hypertensives (thiazides, ARBs, calcium-channel blockers, ACE-Inhibitors, diuretics, Fixed Dose Combinations (e.g. lisinopril-hydrochlorothiazide), epinephrine injections & centrally acting agents), anti-diabetics (metformin) and bronchodilators (salbutamol)

**Supply chain structures and transportation**

The pharmacist is the principal person responsible for managing the ordering of the facility’s medical supply. The national medical store is the main source for routine pharmaceutical supplies and the facility has to arrange the mechanisms of transportation to deliver product to the facility. For routine medications, private suppliers deliver stock to the facility and the typical time interval between product ordering and delivery is usually less than 2 weeks. Push distribution, in which a higher level send supplies and medication, is the main mechanism of determining the facility’s resupply need and there is no specific formula for determining the quantity needed for resupply.

# PHC 9

**Health facility description and personnel**

This is a Comprehensive Health Centre situated at the . It currently serves a population of about 44,000 as against recommended 10,000 – 20,000 population coverage. It has 3 full-time Medical Doctor and 7 nurses of which 2 are part-time. The facility also has 1 part-time Junior Community Health Extension Worker (JCHEW), there are 1 full time Community Health Officers (CHO) and 2 (1 fulltime and 1 part-time) Community Health Extension Worker (CHEW) (Table 1). This PHC runs a shift duty so that there is at least a medical doctor on duty throughout the 24 hours. It is also referred to as flagship centre where lower level of PHCs can refer patient to.

**Patients access to facility**

The PHC operates a 24-hour services and patients can access the facilities by a follow up appointment or walk in whenever there is a need. All basic primary health care services such as immunization and treatment of minor ailments are being offered within the facility. Use of paper file is the only means of keeping patients’ records and the patients’ information are kept by the facility only. Apart from labour-ward/maternity beds, the PHC has only 1 bed provision for in-patient’s admission (observation mainly); and attended to 1556 patients in the month preceding assessment. Of these patients, 12 were hypertensive and 9 was diabetic.

**Basic amenities**

The facilities main supply of electricity is the national grid but uses a generator as back up electricity source. The facility experiences more than 2 hours interruption of electricity supplies. Water piped within the facility is the main source of water and there is a functioning flushed toilet type in the PHC. Final disposal of waste is done by removal from offsite by the medical branch of State Waste Management Agency. The facility has a functional computer but no dedicated landline telephone or private telephone. However, individual staff has their personal phone through which they have access to internet or private email.

**Service delivery for NCDs**

The facility screens, diagnose, prescribe and dispense initial and follow up treatment for high blood pressure and diabetes cases. It also provides long term care for these groups of patients. Some staff of the facilities have received in-service training for management of patients with hypertension and diabetics within the last 2 years. CHWs are also involved in the management of NCDs cases. The facility refers NCDs cases to the nearest secondary health facilities such as General Hospital for some reasons such as lack of BP control despite 3 drug therapy. There is a functional ambulance available to convey referred patients, at no cost to the patients or their relatives. However, the facility has no national guideline or treatment algorithm for the management of NCDs.

Though there is no information, education and communication (IEC) materials available, the facility conducts counselling and education for patients and their relatives on NCDs risk factors. The facility does not also receive any community support concerning NCDs services.

**Basic NCDs equipment, diagnostic kits and drugs**

The facility has at least one functional sphygmomanometer, stethoscope, adult weighing scale, glucometer, thermometer and measuring tape. There are also test kits to check for urine glucose, protein, ketone bodies but not for albumin.

The facility also has in stock some basic NCDs medications such as anti-hypertensives (thiazides, calcium-channel blockers, ACE-Inhibitors, diuretics, vasodilators, epinephrine injections & centrally acting agents), anti-diabetics (metformin) and bronchodilators (salbutamol)

**Supply chain structures and transportation**

The pharmacist is the principal person responsible for managing the ordering of the facility’s medical supply. The national medical store is the main source for routine pharmaceutical supplies and the facility has to arrange the mechanisms of transportation to deliver product to the facility. For routine medications, private suppliers deliver stock to the facility and the typical time interval between product ordering and delivery is usually less than 2 weeks. Push distribution, in which a higher level send supplies and medication, is the main mechanism of determining the facility’s resupply need and there is no specific formula for determining the quantity needed for resupply.

# PHC 10

**Health facility description and personnel**

This is a Comprehensive Health Centre situated within the Southern region. It currently serves a population of about 47,808 as against recommended 10,000 – 20,000 population coverage. It has 4 full-time Medical Doctor and 9 nurses of which 3 are part-time. The facility also has 2 part-time Junior Community Health Extension Worker (JCHEW), there are 4 full time Community Health Officers (CHO) and 3 fulltime Community Health Extension Worker (CHEW). This PHC runs a shift duty so that there is at least a medical doctor on duty throughout the 24 hours. It is also referred to as flagship centre where lower level of PHCs can refer patient to.

**Patients access to facility**

The PHC operates a 24-hour services and patients can access the facilities by a follow up appointment or walk in whenever there is a need. All basic primary health care services such as immunization and treatment of minor ailments are being offered within the facility. Use of paper file is the only means of keeping patients’ records and the patients’ information are kept by the facility only. Apart from labour-ward/maternity beds, the PHC has no bed for in-patient’s admission; and attended to 1934 patients in the month preceding assessment. Of these patients, 12 were hypertensive and none was diabetic.

**Basic amenities**

The facilities main supply of electricity is the national grid but uses a generator as back up electricity source. The facility experiences more than 2 hours interruption of electricity supplies. Water piped within the facility is the main source of water and there is a functioning flushed toilet type in the PHC. Final disposal of waste is done by removal from offsite by the medical branch of the State Waste Management Agency. The facility has a functional computer but no dedicated landline telephone or private telephone. However, individual staff has their personal phone through which they have access to internet or private email.

**Service delivery for NCDs**

The facility screens, diagnose, prescribe and dispense initial and follow up treatment for high blood pressure and diabetes cases. It also provides long term care for these groups of patients. Some staff of the facilities have received in-service training for management of patients with hypertension and diabetics within the last 2 years. CHWs are also involved in the management of NCDs cases. The facility refers NCDs cases to the nearest secondary health facilities such as a General Hospital and State University Teaching Hospital for some reasons such as lack of BP control despite 3 drug therapy. There is a functional ambulance available to convey referred patients, at no cost to the patients or their relatives. However, the facility has no national guideline or treatment algorithm for the management of NCDs.

Though there is no information, education and communication (IEC) materials available, the facility conducts counselling and education for patients and their relatives on NCDs risk factors. The facility does not also receive any community support concerning NCDs services.

**Basic NCDs equipment, diagnostic kits and drugs**

The facility has at least one functional sphygmomanometer, stethoscope, adult weighing scale, glucometer, thermometer and measuring tape. There are also test kits to check for urine glucose, protein, ketone bodies but not for albumin.

The facility also has in stock some basic NCDs medications such as anti-hypertensives (thiazides, ARBs, calcium-channel blockers, ACE-Inhibitors, diuretics, epinephrine injections & Fixed Dose Combinations e.g. lisinopril-hydrochlorothiazide)), anti-diabetics (metformin) and bronchodilators (salbutamol)

**Supply chain structures and transportation**

The pharmacist is the principal person responsible for managing the ordering of the facility’s medical supply. The national medical store is the main source for routine pharmaceutical supplies and the facility has to arrange the mechanisms of transportation to deliver product to the facility. For routine medications, private suppliers deliver stock to the facility and the typical time interval between product ordering and delivery is usually less than 2 weeks. Push distribution, in which a higher level send supplies and medication, is the main mechanism of determining the facility’s resupply need and there is no specific formula for determining the quantity needed for resupply.

# PHC 11

**Health facility description and personnel**

This is a Comprehensive Health Centre situated within the Southern region. It currently serves a population of about 47,650 as against recommended 10,000 – 20,000 population coverage. It has 2 full-time Medical Doctors and 11 nurses of which 1 are part-time. The facility also has 1 full-time Junior Community Health Extension Worker (JCHEW), there are 13 Community Health Extension Worker (CHEW) of which 7 are part-time but there are no Community Health Officers (CHO) (Table 1). This PHC only has doctor on duty for weekdays dayshift (8am – 4pm) of the 24 hours service period.

**Patients access to facility**

The PHC operates a 24-hour services and patients can access the facilities by a follow up appointment or walk in whenever there is a need. All basic primary health care services such as immunization and treatment of minor ailments are being offered within the facility. Use of paper file is the only means of keeping patients’ records and the patients’ information are kept by the facility only. Apart from labour-ward/maternity beds, the PHC has 12 bed for in-patient’s admission; and attended to 2963 patients in the month preceding assessment. Of these patients, 18 were hypertensive and none was diabetic.

**Basic amenities**

The facilities main supply of electricity is the national grid but uses a generator as back up electricity source. The facility experiences more than 2 hours interruption of electricity supplies. Water piped within the facility is the main source of water and there is a functioning flushed toilet type in the PHC. Final disposal of waste is done by removal from offsite and taken to another protected site. The facility has a functional computer and private telephone but no dedicated landline telephone. However, individual staff has their personal phone through which they have access to internet or private email.

**Service delivery for NCDs**

The facility screens, diagnose, prescribe and dispense initial and follow up treatment for high blood pressure and diabetes cases. It also provides long term care for these groups of patients. No staff of the facilities have received in-service training for management of patients with hypertension and diabetics within the last 2 years. CHWs are also involved in the management of NCDs cases. The facility refers NCDs cases to the nearest secondary health facilities for some reasons such absolute BP level of 220/120mmHg and as lack of BP control despite 3 drug therapy. There is a functional ambulance available to convey referred patients, at no cost to the patients or their relatives. However, the facility has no national guideline However, the facility has no national guideline or but uses PACK guideline as treatment algorithm for the management of NCDs.

Though there is no information, education and communication (IEC) materials available, the facility conducts counselling and education for patients and their relatives on NCDs risk factors. The facility does not also receive any community support concerning NCDs services.

**Basic NCDs equipment, diagnostic kits and drugs**

The facility has at least one functional sphygmomanometer, stethoscope, adult weighing scale, glucometer, thermometer and measuring tape. There are also test kits to check for urine glucose, protein, ketone bodies but not for albumin.

The facility also has in stock some basic NCDs medications such as anti-hypertensives (calcium-channel blockers, ACE-Inhibitors, diuretics, epinephrine injections & centrally acting agents), and bronchodilators (salbutamol) but no anti-diabetics (e.g. metformin)

**Supply chain structures and transportation**

The pharmacist is the principal person responsible for managing the ordering of the facility’s medical supply. The private suppliers are the main source for routine pharmaceutical supplies medications to the facility. The private suppliers deliver stock to the facility and the typical time interval between product ordering and delivery is usually less than 2 weeks. Pull distribution, in which the facility make request, is the main mechanism of determining the facility’s resupply need and there is no specific formula for determining the quantity needed for resupply.

# PHC 12

**Health facility description and personnel**

This is a Comprehensive Health Centre situated within the Southern region, It currently serves a population of about 3,560 as against recommended 10,000 – 20,000 population coverage. It has 1 full-time Medical Doctor and 3 nurses. The facility also has 2 full-time Junior Community Health Extension Worker (JCHEW) and 4 Community Health Extension Worker (CHEW) of which 1 is part-time staff but there are no Community Health Officers (CHO) (Table 1). This PHC only has doctor on duty for weekdays dayshift (8am – 4pm) of the 24 hours service period.

**Patients access to facility**

The PHC operates a 24-hour services and patients can access the facilities by a follow up appointment or walk in whenever there is a need. Most basic primary health care services such as immunization and treatment of minor ailments are being offered within the facility. Use of paper file is the only means of keeping patients’ records and the patients’ information are kept by the facility only. Apart from labour-ward/maternity beds, the PHC has 8 bed for in-patient’s admission; and attended to 332 patients in the month preceding assessment. Of these patients, 15 were hypertensive and none was diabetic.

**Basic amenities**

The facilities main supply of electricity is the national grid but uses a generator as back up electricity source. The facility experiences more than 2 hours interruption of electricity supplies. Water piped within the facility is the main source of water and there is a functioning flushed toilet type in the PHC. Final disposal of waste is done by removal from offsite and taken to another protected site. The facility has a functional computer and private telephone but no dedicated landline telephone. However, individual staff has their personal phone through which they have access to internet or private email.

**Service delivery for NCDs**

The facility screens, diagnose, prescribe and dispense initial and follow up treatment for high blood pressure and diabetes cases. It also provides long term care for these groups of patients. Some staff of the facilities have received in-service training for management of patients with hypertension and diabetics within the last 2 years. CHWs are also involved in the management of NCDs cases. The facility refers NCDs cases to the nearest secondary health facilities for some reasons such absolute BP level of 220/120mmHg and as lack of BP control despite 3 drug therapy. There is a functional ambulance available to convey referred patients, at no cost to the patients or their relatives. However, the facility has no national guideline or but uses PACK guideline as treatment algorithm for the management of NCDs.

Though there is no information, education and communication (IEC) materials available, the facility conducts counselling and education for patients and their relatives on NCDs risk factors. The facility does not also receive any community support concerning NCDs services.

**Basic NCDs equipment, diagnostic kits and drugs**

The facility has at least one functional sphygmomanometer, stethoscope, adult weighing scale, glucometer, thermometer and measuring tape. There are also test kits to check for urine glucose, protein, ketone bodies but not for albumin.

The facility also has in stock some basic NCDs medications such as anti-hypertensives (Thiazides, calcium-channel blockers, ACE-Inhibitors, diuretics, Fixed Dose Combinations (e.g. lisinopril-hydrochlorothiazide), epinephrine injections & centrally acting agents) but no bronchodilators (salbutamol) or anti-diabetics (e.g. metformin)

**Supply chain structures and transportation**

The pharmacist technician is the principal person responsible for managing the ordering of the facility’s medical supply. The private suppliers are the main source for routine pharmaceutical supplies medications to the facility. The facility arranges the mechanisms of transportation to deliver product to the facility and the typical time interval between product ordering and delivery is usually less than 2 weeks. Pull distribution, in which the facility make request, is the main mechanism of determining the facility’s resupply need and there is no specific formula for determining the quantity needed for resupply.

# PHC 13

**Health facility description and personnel**

This is a Comprehensive Health Centre situated at the Akure-South Local Government Area, Ondo State. It currently serves a population of about 28,929 as against recommended 10,000 – 20,000 population coverage. It has 4 nurses of which 1 is a part-time staff but no Medical Doctor. The facility also has 1 full-time Junior Community Health Extension Worker (JCHEW), 6 Community Health Extension Worker (CHEW) of which 3 are part-time but there are no Community Health Officers (CHO) (Table 1).

**Patients access to facility**

The PHC operates a 24-hour services and patients can access the facilities by a follow up appointment or walk in whenever there is a need. Most basic primary health care services such as immunization and treatment of minor ailments are being offered within the facility. Use of paper file is the only means of keeping patients’ records and the patients’ information are kept by the facility only. Apart from labour-ward/maternity beds, the PHC has 8 bed for in-patient’s admission; and attended to 660 patients in the month preceding assessment. Of these patients, 4 were hypertensive and none was diabetic.

**Basic amenities**

The facilities main supply of electricity is fuel-powered generator and only uses the national grid as back up electricity source whenever it’s available. The facility experiences more than 2 hours interruption of electricity supplies. Water piped within the facility is the main source of water and there is a functioning flushed toilet type in the PHC. Final disposal of waste is done by removal from offsite and taken to another protected site. The facility has private telephone but no functional computer or dedicated landline telephone.

**Service delivery for NCDs**

The facility screens, diagnose, prescribe and dispense initial but not follow up treatment for high blood pressure and diabetes cases. It does not also provide long term care for these groups of patients. No staff of the facilities have received in-service training for management of patients with hypertension and diabetics within the last 2 years. CHWs are also involved in the management of NCDs cases. The facility refers NCDs cases to the nearest secondary health facilities for some reasons such absolute BP level of 140/90mmHg and as lack of BP control despite 3 drug therapy. There is no functional ambulance available to convey referred patients, and the patients or their relatives makes arrangement whenever they are referred. The facility does not have a national guideline or treatment algorithm for the management of NCDs.

Though there is no information, education and communication (IEC) materials available, the facility conducts counselling and education for patients and their relatives on NCDs risk factors. The facility does not also receive any community support concerning NCDs services.

**Basic NCDs equipment, diagnostic kits and drugs**

The facility has at least one functional sphygmomanometer, stethoscope, adult weighing scale, glucometer, thermometer and measuring tape. There are also test kits to check for urine glucose, protein, ketone bodies but not for albumin.

The facility also has in stock some basic NCDs medications such as anti-hypertensives (calcium-channel blockers & centrally acting agents), but no bronchodilators (salbutamol) or anti-diabetics (e.g. metformin)

**Supply chain structures and transportation**

The Matron-in-charge of the facility is the principal person responsible for managing the ordering of the facility’s medical supply. The private suppliers are the main source for routine pharmaceutical supplies medications to the facility. The private suppliers deliver stock to the facility and the typical time interval between product ordering and delivery is usually less than 2 weeks. Pull distribution, in which the facility make request, is the main mechanism of determining the facility’s resupply need and there is no specific formula for determining the quantity needed for resupply.
