# Supplementary material for: The organisation of primary health care service delivery for non-communicable diseases in Nigeria: A case-study analysis
Source: PLOS Glob Public Health. 2022 Jul 1;2(7):e0000566. doi: 10.1371/journal.pgph.0000566 (PMC10021956; doi:10.1371/journal.pgph.0000566)
Supplement: Attachment — Submitted filename: Response to Reviewers Comment_Final.docx [file pgph.0000566.s004.docx]

The George Institute for Global Health,

Level 5, 1 King St, Newtown, NSW 2042, Australia

10^th^ May, 2022

**Dear Roopa Shivashankar,**
Academic Editor
PLOS Global Public Health

**Dear Editor in Chief,**

**Re: Revision required [PGPH-D-22-00322: *“The organisation of primary health care service delivery for non-communicable diseases in Nigeria: A case-study analysis” –* Response to reviewers’ comments**
Thank you for reviewing our manuscript and giving us the opportunity to revise it further. We have responded to the comments raised by the reviewers (below) and revised the manuscript accordingly.

**Journal requirement:** We have responded to all requirements (see below). We did not cite any paper that have been retracted papers, and have updated the references list.

Also submitted along with this response are the ‘clean’ and ‘tracked changes’ copies of the manuscript.

Please do not hesitate to contact me should you require any further clarification.

Yours sincerely,

**Whenayon Simeon Ajisegiri**

Tel.: +61469238057

E-mail: wajisegiri@georgeinstitute.org.au

**RESPONSE TO JOURNAL REQUIREMENTS**

1. Please include a complete copy of PLOS’ questionnaire on inclusivity in global research in your revised manuscript. Our policy for research in this area aims to improve transparency in the reporting of research performed outside of researchers’ own country or community. The policy applies to researchers who have travelled to a different country to conduct research, research with Indigenous populations or their lands, and research on cultural artefacts. The questionnaire can also be requested at the journal’s discretion for any other submissions, even if these conditions are not met.  Please find more information on the policy and a link to download a blank copy of the questionnaire here: https://journals.plos.org/plosone/s/best-practices-in-research-reporting. Please upload a completed version of your questionnaire as Supporting Information when you resubmit your manuscript.

**Response:** A completed version of the of PLOS’ questionnaire on inclusivity in global research has been uploaded as Supporting Information

2. Your co-authors:
   David Peiris -dpeiris@georgeinstitute.org
   Rohina Joshi -rohina.joshi@unsw.edu.au
   ,have not confirmed authorship of the manuscript. We have resent them the authorship confirmation email; however please check that the above email address for them is correct and follow up personally to ensure they confirm.

Please note that we cannot proceed your manuscript  until we have received confirmations from all co-authors.

**Response:** I have contacted them, and they have confirmed their authorship.

3. Please include details in the Funding Information section in the system. It should matched with the Financial Disclosure Statement.

**Response:** This has been included

4. We have noticed that you have uploaded supporting information, but you have not included a list of legends.  Please add a full list of legends for all supporting information files (including figures, table and data files) after the references list.

**Response:** A full list of legends for all supporting information files has been added to the manuscript (after the references list).

5. In the online submission form, you indicated that "All relevant data contributing to the findings are within the study. The raw data (transcript from qualitative study) are stored on a secure network and cannot be made publicly available in order to protect participant confidentiality (Making them publicly available also contradict the terms contained in the ethical approval for the study).". All PLOS journals now require all data underlying the findings described in their manuscript to be freely available to other researchers, either 1. In a public repository, 2. Within the manuscript itself, or 3. Uploaded as supplementary information.
   This policy applies to all data except where public deposition would breach compliance with the protocol approved by your research ethics board. If your data cannot be made publicly available for ethical or legal reasons (e.g., public availability would compromise patient privacy), please explain your reasons by return email and your exemption request will be escalated to the editor for approval. Your exemption request will be handled independently and will not hold up the peer review process, but will need to be resolved should your manuscript be accepted for publication. One of the Editorial team will then be in touch if there are any issues.

**Response:** Thanks for the update. All raw quantitative data from the surveys have been anonymised and uploaded as supplementary files (MS Excel and MS Word documents). We request exemption from the editor for access to the raw qualitative data (transcripts) as this would compromise the confidentiality of participants and would be in breach of ethics requirements.

**RESPONSE TO REVIEWER’S COMMENTS**

**Reviewer #1:**

The paper highlights some very important issues about the PHC system in Nigeria. It needs minor edits and additions.

A. Please clarify this quote- 'consequently, NCD care at the PHC level is mainly handled by community health workers (CHWs), who are not generally lack sufficient training in NCD management and prevention [12]'.

**Response:**
We acknowledged there are some typo errors in the statement. It has been corrected to read *‘Consequently, NCD care at the PHC level is mainly handled by community health workers (CHWs), whose training and skills are generally considered insufficient for NCD management and prevention [14].’* **[Page 4, paragraph 2 under Introduction section]**

B. The issue of referral linkages was highlighted but not discussed- PHC should be the entry point into the health system and so this should be addressed.
   **Response:**
   We completely agree that ‘the PHC is the entry point into the health system’. We discussed referral linkage under the sub-section “Inadequate health workforce at the PHC, and the perception of PHC as an inferior component of the health system”. Our study primarily examined referral linkage between PHC and secondary health facilities, and we highlighted the existence of this established linkages as an enabler. However, we noted that this referral linkage is weakened if community members and/or personnel at secondary health facilities perceive PHC to be inferior care.

In order to more strongly emphasize the point raised, we have included the statement, “*Being the closest entry point of the community into the formal health system, the PHC sector is best placed to reach the ‘last mile population’ who predominantly reside in rural areas”* to the introductory paragraph that discussed issues on referral linkages. **[Page 22 paragraph 2 under Discussion section]**

C. It is worth discussing that the study has shown that different models of PHC (CHEW vs Physician led) and different contexts (North vs South; Rural vs Urban) were assessed therefore further contextualisation of the findings will make the recommendations more useful by policy makers.

**Response:**
Thanks for pointing this out. The paragraph below has been added to the discussion section to address this important issue.

*“Our study shows different models of PHC (CHEW vs Physician led) which vary significantly by context: northern states vs southern states; and rural communities vs urban communities. Our findings can inform the ongoing efforts of state PHC agencies to re-organise NCD service delivery in a way that not only reflects their greater capacity relative to local governments, but also reflects greater contextualisation to local needs and capacity.”* **[Page 26 paragraph 3 under discussion section]**

D. Some background on the policy direction of the PHC system in Nigeria will also provide some useful background for the readers of the paper.

**Response:**
Thanks. We have added some background information to the ‘Nigeria PHC system’ discussed in the introduction section.

*‘Between 1986 to 1990, the PHC experienced several reforms including its expansion to all LGAs and the creation of colleges of health technology for the training of community health workers [8]. In 2014, the National Health Act placed the PHCs under the local government authorities [9]. This however created a bottleneck for PHC funding as federal laws are not binding on the states in a decentralized health system [8]’* **[Page 3 paragraph 2 under Introduction section]**

We also discussed important policy direction for the PHC system in the discussion section as stated below:

*‘An important policy direction for PHC in Nigeria is the development of new state-level PHC agencies to take over PHC governance from both local and national governments. On the one hand, the state-level PHC agencies will centralise PHC management within a state by taking over responsibilities from local government councils – given their weak financial and technical capacity in most states. On the other hand, state-level PHC agencies may also take over direct policy guidance from the national PHC agency – thereby better tailoring PHC policies to local needs and circumstances.’* **[Page 26 paragraph 2 under discussion section]**

**Reviewer #2:**

This is a well written paper highlighting the major challenges to manage non-communicable diseases in Nigeria at primary health care level. There are few suggestions which authors may consider.

A. Justification for selecting health workers who have worked for a minimum of three
   months at the facility in the study
   **Response:**
   Because staff turnover can be an issue, we sought perspectives from health workers that had a detailed understanding of facility operations. We have added the following text to clarify this: *“This is to ensure that participants had worked for a sufficient duration to have a detailed understanding of how their facilities operate****”* [Page 8 paragraph 1 under Methods section]**
B. Insufficient staffing is the major issue in result section, but it is not discussed in discussion, nor it is part of figure 2.

**Response:**
A paragraph addressing ‘insufficient staffing’ has been added to the discussion section, under the modified sub-section “**Inadequate health workforce at the PHC, and the perception of PHC as an inferior component of the health system”.**

The paragraph reads  *‘An adequate health workforce in terms of supply and skill mix is essential for effective NCD service delivery. A major barrier observed in this study is that most PHCs facilities do not meet the minimum staffing requirements stipulated by the Ward Health System within which each PHC operates [10]. This persistent workforce shortage combined with a large pool of volunteer community health workers likely reflects limited funding allocations for the PHC sector. National and subnational governments need to accelerate and scale up staff recruitment combined with appropriate skills training[40], and proportionate distribution to areas of relative low workforce density [41]. This requires development of evidence-informed workforce policies, effective deployment mechanisms [41] and increased funding allocation.’*  **[Page 22 paragraph 2 under Discussions section]**

**‘Insufficient training’ has also been added to figure 2 (under ‘health workforce’ heading)**

C. Community linkage is another enabler from, and Performance-Based Financing (PBF) program are another enabler discussed which can be added in figure 2 under referral heading
   **Response:**
   Thanks for the recommendation. These two enablers have been added to figure 2 **(under ‘referral’ heading)**
